# Beyond BMI: Ultrasound-Detected Visceral Adiposity as a Predictor of Early Cardiometabolic Dysfunction in Youth with Type 1 Diabetes

**DOI:** 10.3390/children13010124

**Published:** 2026-01-14

**Authors:** Sukriye Ozde, Gulsah Akture, Mehmet Ali Ozel, Fatma Yavuzyilmaz, Cem Ozde, Osman Kayapinar, Ilknur Arslanoglu

**Affiliations:** 1 Department of General Pediatric, Faculty of Medicine, Duzce University, Duzce 81620, Turkey; sukriyeozde@duzce.edu.tr; 2 Department of Cardiology, Faculty of Medicine, Duzce University, Duzce 81620, Turkey; gulsahakture@duzce.edu.tr (G.A.); cemozde@duzce.edu.tr (C.O.); 3 Department of Radiology, Faculty of Medicine, Duzce University, Duzce 81620, Turkey; mehmetaliozel@duzce.edu.tr (M.A.O.); fatmayavuzyilmaz@duzce.edu.tr (F.Y.); 4 Department of Pediatric Endocrinology, Faculty of Medicine, Duzce University, Duzce 81620, Turkey; ilknurarslanoglu@duzce.edu.tr

**Keywords:** type-1 diabetes mellitus, visceral adiposity

## Abstract

**Highlights:**

**What are the main findings? **

• Perirenal and epicardial adipose tissue thicknesses are significantly increased in children and adolescents with type 1 diabetes mellitus, independent of overall body mass index.
• Higher visceral adipose tissue thickness is associated with reduced insulin sensitivity, systemic inflammation, and increased carotid intima–media thickness, indicating early subclinical atherosclerosis.

**What are the implications of the main findings? **

• Ultrasonographic assessment of perirenal and epicardial fat provides a simple, noninvasive tool for early cardiometabolic risk stratification in pediatric type 1 diabetes.
• Visceral adiposity–based imaging markers may help identify high-risk children who could benefit from earlier preventive cardiovascular interventions beyond glycemic control alone.

**Abstract:**

**Background:** Visceral adiposity has emerged as a clinically relevant determinant of early cardiometabolic dysfunction in pediatric type 1 diabetes mellitus (T1DM), yet its assessment remains underutilized in routine practice. This study evaluated ultrasonographically measured epicardial adipose tissue thickness (EATT) and perirenal adipose tissue thickness (PrATT) as markers of metabolic risk, insulin sensitivity, and subclinical atherosclerosis in children and adolescents with T1DM. **Methods:** This cross-sectional study included 150 participants with T1DM and 152 age- and sex-matched healthy controls. Anthropometric data, biochemical parameters, hepatic steatosis grade, and insulin sensitivity indices (eGDR) were collected. EATT and PrATT were measured via standardized echocardiographic and abdominal ultrasonographic protocols. Carotid intima–media thickness (cIMT) was assessed as an indicator of subclinical atherosclerosis. Correlation and multivariable logistic regression analyses were performed to identify independent predictors of T1DM status and cardiometabolic risk. **Results:** Children with T1DM exhibited significantly higher PrATT and EATT values compared with controls (both *p* < 0.05). All eGDR indices were markedly lower in the T1DM group, reflecting reduced insulin sensitivity. PrATT and EATT showed strong or moderate correlations with hsCRP, hepatic steatosis, atherogenic index of plasma, and multiple anthropometric markers. Both visceral fat depots were positively associated with cIMT. Logistic regression identified PrATT, EATT, hsCRP, cIMT, and eGDR-BMI as independent predictors of case status. Subgroup analyses demonstrated more pronounced visceral adiposity and metabolic impairment among participants with BMI ≥85th percentile. **Conclusions:** Ultrasonographically measured PrATT and EATT provide valuable insight into early cardiometabolic risk in youth with T1DM, independent of BMI. Their associations with insulin resistance, inflammation, and subclinical atherosclerosis highlight their potential utility as accessible markers for early risk stratification in pediatric diabetes. Routine incorporation of visceral fat assessment may support earlier identification of high-risk individuals and more targeted preventive strategies.

## 1. Introduction

Adipose tissue is not merely a passive depot for energy storage but a dynamic organ with important endocrine, paracrine, and immunoinflammatory functions [1]. It consists of heterogeneous adipocyte populations—white, brown, and beige—and is organized into several depots with distinct morphology and physiological roles. These tissues contain adipocytes embedded within rich vascular and neural networks, together with preadipocytes, pericytes, and inflammatory cells [1]. In addition to maintaining local and systemic homeostasis, adipose tissue contributes to the pathogenesis of several cardiometabolic and inflammatory disorders [1]. Visceral adiposity is associated with dyslipidemia, insulin resistance, atherosclerosis, and increased mortality. Although dual-energy X-ray absorptiometry and computed tomography allow direct assessment of visceral fat, they are limited by cost, radiation exposure, and accessibility. Therefore, simple surrogate markers that indirectly reflect visceral fat distribution and function are clinically valuable. Given their heterogeneous distribution and diverse metabolic features, the anatomical characteristics of visceral fat depots warrant particular attention.

In humans, most adipose tissue is stored as white adipose tissue in subcutaneous and visceral regions. Visceral adipose tissue is subdivided into discrete compartments, including gonadal, epicardial, retroperitoneal, mesenteric, omental, and perirenal depots. Among these, perirenal adipose tissue (PRAT) has gained increasing attention as a metabolically active visceral fat pad influencing cardiovascular and metabolic regulation [1,2]. PRAT secretes adipokines and cytokines exerting endocrine and paracrine effects, and its thickness correlates strongly with metabolic activity. Ultrasonography allows accurate, accessible, and inexpensive assessment of PRAT thickness, making it a suitable tool for clinical practice [2].

Epicardial adipose tissue (EAT) is another metabolically active visceral depot located between the visceral pericardium and the myocardium [3]. Epicardial adipose tissue contributes directly to myocardial energy homeostasis and functions as a paracardiac reservoir of biologically active mediators capable of influencing vascular tone, immune activity, and inflammatory signaling [3,4]. These adipokines and cytokines can directly affect the myocardium and coronary arteries. Epicardial adipose tissue thickness (EATT), measured by transthoracic echocardiography, is an indirect marker of total EAT volume and benefits from ease of access, low cost, reproducibility, and absence of radiation [4,5].

Type 1 diabetes mellitus (T1DM) typically presents in childhood or adolescence and is characterized by accelerated development of atherosclerotic complications, making it a significant global health concern [6,7]. Despite improvements in diagnosis and therapy, mortality remains two- to eightfold higher than in the nondiabetic population, largely due to cardiovascular disease [6,7]. Robust evidence shows that T1DM is associated with premature and accelerated atherosclerosis. A recent study in young adults demonstrated that early atherosclerotic plaques predicted all-cause mortality and major cardiovascular events independently of traditional risk factors [6]. Early detection of subclinical atherosclerosis is therefore critical in pediatric T1DM.

Children with T1DM exhibit early vascular alterations, including endothelial dysfunction and arterial wall thickening. Carotid intima–media thickness (cIMT) is a validated noninvasive marker of subclinical atherosclerosis and has been shown to predict future cardiovascular events [8]. Elevated cIMT has consistently been reported in pediatric T1DM cohorts, supporting its role as a valuable tool for early cardiovascular risk stratification [8,9,10].

Therefore, the primary aim of this study was to evaluate perirenal adipose tissue thickness (PrATT) and epicardial adipose tissue thickness (EATT) in children and adolescents with T1DM compared with healthy controls, and to examine their relationships with insulin sensitivity (estimated glucose disposal rate, eGDR), cIMT, and composite cardiometabolic indices. We hypothesized that PrATT and EATT would be increased in pediatric T1DM, even in the absence of marked obesity, and that higher values would be associated with reduced insulin sensitivity, greater ectopic fat burden, and early vascular changes.

## 2. Materials and Methods

### 2.1. Study Population

This cross-sectional observational study was conducted at the Pediatric Endocrinology Unit of Düzce University Faculty of Medicine in accordance with the principles of the Declaration of Helsinki (2013 revision). Approval was obtained from the institutional ethics committee, and written informed consent was obtained from the parents or legal guardians of all participants. Assent was also obtained from children aged ≥12 years.

The study enrolled 150 children and adolescents aged 10–22 years with a confirmed diagnosis of T1DM for at least five years. A control group of 152 age- and sex-matched healthy children and adolescents was recruited consecutively from the general pediatric outpatient clinic. T1DM was diagnosed according to the International Society for Pediatric and Adolescent Diabetes (ISPAD) criteria, which include plasma glucose > 200 mg/dL accompanied by classic symptoms, the presence of ketonuria or ketoacidosis, positivity for at least one islet-cell autoantibody, or a reduced C-peptide concentration.

Information on insulin therapy regimen (multiple daily injections or continuous subcutaneous insulin infusion/continuous glucose monitoring–assisted therapy) and metabolic control was retrieved from the hospital’s electronic database. Detailed medical histories and comprehensive physical examinations were performed for all participants.

To minimize confounding, the following exclusion criteria were applied: endocrine disorders other than T1DM; established chronic diabetes complications; autoimmune or rheumatologic diseases; malignancy; chronic allergic diseases; unexplained fever or infection within the preceding month; history of multisystem inflammatory syndrome in children (MIS-C) related to severe acute respiratory syndrome coronavirus 2 (SARS-CoV-2); past or current use of systemic glucocorticoids, cytotoxic agents, or lipid-lowering drugs; irregular three-monthly follow-up visits; or incomplete electronic medical records.

### 2.2. Anthropometric Measurements and Derived Indices

Anthropometric assessments were performed by trained investigators using standardized procedures. Standing height was measured to the nearest 0.1 cm with a wall-fixed stadiometer, and body weight was recorded to the nearest 0.1 kg using calibrated electronic scales. All measurements were obtained while participants were barefoot and wearing light clothing. Measurement devices were calibrated before the start of the study and periodically throughout the data collection period.

Waist and hip circumferences were obtained in the standing position using a non-elastic measuring tape during normal breathing. Waist circumference was measured midway between the inferior margin of the last palpable rib and the iliac crest, whereas hip circumference was measured at the level of the greatest gluteal circumference. Waist-to-hip ratio (WHR) was calculated as waist circumference divided by hip circumference, and waist-to-height ratio (WHtR) as waist circumference divided by standing height.

Insulin sensitivity was estimated using the estimated glucose disposal rate (eGDR), a validated surrogate marker of whole-body insulin sensitivity [11,12]. Hypertension status was treated as a binary variable (present = 1, absent = 0), and glycated hemoglobin (HbA1c) was expressed as a percentage. Three established equations were used to calculate eGDR based on different anthropometric parameters:

• **eGDR–WHR** = 24.31 − (12.22 × WHR) − (3.29 × HTN) − (0.57 × HbA1c)
• **eGDR–WC** = 21.158 − (0.09 × WC [cm]) − (3.407 × HTN) − (0.551 × HbA1c)
• **eGDR–BMI** = 19.02 − (0.22 × BMI [kg/m^2^]) − (3.26 × HTN) − (0.61 × HbA1c)

Body mass index (BMI) was calculated as weight divided by height squared (kg/m^2^) and subsequently converted to age- and sex-specific percentile values using national reference data for Turkish children and adolescents [13]. Participants younger than 18 years were categorized as underweight (<5th percentile), normal weight (5th to <85th percentile), overweight (85th to <95th percentile), or obese (≥95th percentile) [14]. For individuals aged 18 years or older, standard adult BMI classification criteria were applied. Age was calculated in decimal years as the interval between date of birth and date of examination.

### 2.3. Echocardiographic Assessment and Evaluation of EATT

Comprehensive transthoracic echocardiographic examinations were carried out in all participants, who were positioned in the left lateral decubitus posture. Standard apical four-chamber and two-chamber views, together with parasternal long- and short-axis views, were obtained during brief end-expiratory breath holds. Image acquisition was performed across three consecutive cardiac cycles using a phased-array transducer operating at 3.0–4.5 MHz (Vivid 7 Pro, GE Healthcare, Horten, Norway), with continuous lead II electrocardiographic monitoring throughout the procedure.

Image acquisition and primary measurements were independently performed by two experienced cardiologists who were unaware of the participants’ clinical and laboratory characteristics. Offline image analysis was subsequently conducted by two additional cardiologists who were likewise blinded to group allocation. Measurement reproducibility was assessed by calculating the coefficient of variation, defined as the standard deviation of the differences between repeated measurements divided by their mean and expressed as a percentage. Both intraobserver and interobserver variability were below 5%, indicating high measurement reliability.

Epicardial adipose tissue thickness (EATT) was measured according to the American Society of Echocardiography (ASE) guidelines [4]. To obtain the most accurate measurement of EATT over the right ventricle, standard parasternal long-axis and short-axis views were obtained from 2D images with optimal probe orientation. Epicardial adipose tissue was identified on echocardiography as the sonolucent layer located between the myocardial surface and the visceral layer of the pericardium. Given that epicardial fat thickness decreases during diastole as a result of myocardial compression, measurements were obtained at end-systole. Epicardial fat thickness was assessed by placing a vertical caliper on the free wall of the right ventricle, with the aortic annulus serving as the primary anatomical landmark. In the parasternal short-axis view at the mid-ventricular level, the maximal epicardial adipose tissue thickness was measured along the central axis of the ultrasound beam, perpendicular to the interventricular septum, at the level corresponding to the mid-chordal plane and papillary muscle tips. For each participant, measurements from three consecutive cardiac cycles were averaged and used for analysis.

### 2.4. Ultrasonographic Measurement of Perirenal Adipose Tissue Thickness

All subjects were evaluated after at least six hours of fasting, with an empty bladder, in the supine position. Ultrasonographic examinations were performed by a radiologist with >10 years of experience in abdominal ultrasonography. Measurements were obtained using GE Logiq E9 (GE Healthcare, Milwaukee, WI, USA) and Philips Affiniti 70 (Philips Healthcare, Andover, MA, USA) ultrasound systems with a 3.5–5 MHz convex abdominal transducer.

Participants lay in the supine position and, when necessary, a slight lateral decubitus position to improve the acoustic window. Each measurement was taken while the patient briefly held their breath during normal tidal breathing. The kidneys were imaged in the longitudinal (sagittal) plane. Perirenal adipose tissue thickness was measured at the site where the most prominent fat pad was visible in the posterolateral region closest to the renal hilum. The measurement was taken through the hyperechoic layer between the outer capsule of the renal cortex and the inner border of Gerota’s fascia [15]. Pressure on the probe was kept minimal to avoid compressing the fat layer.

Measurements were performed separately for each kidney using the same technique. Three consecutive measurements were recorded for each kidney. The arithmetic mean of the three measurements was calculated for each kidney; then the mean of the right and left kidney values was used as the final perirenal adipose tissue thickness (PrATT) for each patient. Depth, focus, and gain settings were standardized before each session. All measurements were performed in a single-blind manner, with no access to clinical data. Intraobserver variability was <5%.

### 2.5. Laboratory Procedures and Biochemical Analyses

After institutional ethical clearance had been obtained, morning blood samples were drawn from fasting participants as part of their scheduled follow-up assessments. Control specimens were collected from healthy children who consented to participate during scheduled outpatient visits.

HbA1c concentrations were measured in the Clinical Biochemistry Laboratory of Düzce University Medical Faculty Hospital using high-performance liquid chromatography (Variant, Bio-Rad, Richmond, CA, USA). The reference interval was 4.1–6.4%, with an intra-assay coefficient of variation < 3%.

All biochemical analyses were conducted in the same certified laboratory under standardized conditions. Routine serum biochemical parameters were analyzed using an automated Cobas e602 platform (Roche Diagnostics, Berlin, Germany). High-sensitivity C-reactive protein concentrations were determined by nephelometric methods on a Cobas c702 analyzer (Roche Diagnostics). Fasting plasma glucose levels were assessed using an enzymatic assay based on the hexokinase method. Total cholesterol concentrations were measured enzymatically using a colorimetric technique, while high-density lipoprotein cholesterol was quantified with an accelerator-assisted selective detergent method. Triglyceride levels were determined via an enzymatic reaction employing glycerol-3-phosphate oxidase, and low-density lipoprotein cholesterol was estimated using the Friedewald formula. Hematological parameters were obtained with an automated LH500 hematology analyzer (Beckman Coulter, Budapest, Hungary). Plasma fibrinogen concentrations were measured using the Clauss coagulation method on a Diagon Coag XL analyzer (Budapest, Hungary). Erythrocyte sedimentation rate and serum ferritin levels were assessed using routinely applied laboratory techniques.

The atherogenic index of plasma (AIP) was calculated as the base-10 logarithm of the ratio between triglyceride and HDL cholesterol concentrations [log_10_(TG/HDL)]. Based on established thresholds, AIP values were classified as indicating low (<0.11), intermediate (0.11–0.21), or high (>0.21) cardiovascular risk.

### 2.6. Assessment of Cumulative Glycemic Exposure

HbA1c levels for each child with T1DM had been measured every three months since diagnosis in the same laboratory using high-performance liquid chromatography (Bio-Rad Variant; reference range 4.1–6.4%, intra-assay coefficient of variation < 3%). Historical HbA1c data were extracted from electronic records.

Cumulative glycemic exposure was quantified using a method adapted from Orchard et al. [16] and refined by Margeirsdottir et al. [17], which captures both the magnitude and duration of hyperglycemia. Briefly, the number of months between diagnosis and the first HbA1c measurement was multiplied by the amount (percentage points) by which that HbA1c value exceeded the upper limit of normal (6.4%). The same calculation was repeated for each successive interval between HbA1c measurements, and all interval values were summed to obtain total glycemic load, expressed in HbA1c-unit–months. This approach integrates chronic hyperglycemic burden over time and has been shown to predict long-term cardiovascular and microvascular risk more accurately than mean HbA1c alone.

### 2.7. Carotid Intima–Media Thickness Measurement

Carotid intima–media thickness was assessed by high-resolution B-mode ultrasonography in all participants. Examinations were performed by a single experienced sonographer blinded to clinical and laboratory data, using a 7–12 MHz linear-array transducer (GE Vivid or equivalent, Boston, MA, USA). During ultrasonographic evaluation, participants were positioned lying on their backs, with the neck gently extended and the head turned away from the side under examination to optimize vascular imaging. Images were obtained from the far wall of the right and left common carotid arteries, approximately 1 cm proximal to the carotid bulb, following the standardized protocol of the American Society of Echocardiography [18]. Three end-diastolic images were recorded for each side, and measurements were taken at the R-wave of the electrocardiogram to reduce variability. For each artery, mean cIMT was calculated as the average of three measurements within a 10 mm plaque-free segment. Overall cIMT was defined as the mean of the right and left common carotid measurements. Intraobserver reproducibility, assessed in a random subset of participants, showed a coefficient of variation < 5%.

### 2.8. Statistical Analysis

All statistical analyses were performed using Python 3.11 within a Jupyter Notebook libraries. Two-tailed tests were used throughout, and *p* < 0.05 was considered statistically significant. Continuous variables were summarized as mean ± standard deviation or median (interquartile range, IQR) according to their distribution. Normality was assessed with the Shapiro–Wilk test. Between-group comparisons (patients vs. controls) were conducted using Welch’s *t*-test for normally distributed variables or the Mann–Whitney U test for non-normally distributed variables. Categorical variables were compared using the χ^2^ test.

Correlations were evaluated with Pearson or Spearman methods, as appropriate, and visualized using scatter plots with corresponding correlation coefficients. To determine variables independently associated with group membership (type 1 diabetes mellitus versus control), logistic regression analyses were conducted using both univariable and multivariable approaches. Initially, each candidate variable was evaluated separately in univariable models. Variables demonstrating statistical significance at a threshold of *p* < 0.05 were then entered into a multivariable logistic regression framework. A backward elimination strategy was subsequently applied to derive the most parsimonious final model. Regression outcomes are presented as beta coefficients (β), standard errors (SE), Wald z statistics, odds ratios (OR), and corresponding 95% confidence intervals (CI). Lipid profile parameters and hepatic steatosis were analyzed with the same approach; variables that lost significance in the multivariable model are indicated with a dash (–) in the corresponding tables. All analyses and data visualizations were conducted entirely in Python; no additional statistical software (e.g., SPSS) was used.

## 3. Results

A total of 150 adolescents with T1DM and 152 healthy controls were included in the analyses. Demographic and anthropometric characteristics are presented in Table 1. Age and sex distribution did not differ significantly between groups (*p* = 0.201 and *p* = 0.106, respectively). Weight, height, BMI, body surface area, WHtR, WHR, and systolic and diastolic blood pressures were also comparable between patients and controls (all *p* > 0.05). Despite comparable overall anthropometric profiles, waist and hip circumferences were significantly greater in participants with type 1 diabetes mellitus (*p* = 0.010 and *p* = 0.003, respectively), while the BMI percentile demonstrated only a marginal between-group difference (*p* = 0.052). Disease-related parameters (mean HbA1c, disease duration, and glycemic load) were reported only for patients.

Visceral adiposity and cardiometabolic findings are summarized in Table 2. Patients exhibited markedly higher PrATT compared with controls (5.89 ± 1.98 mm vs. 3.72 ± 1.21 mm, *p* < 0.001). EATT was also significantly increased in patients (*p* = 0.022). Hepatic steatosis grade was substantially higher in the T1DM group (*p* < 0.001). Total cholesterol, LDL cholesterol, triglycerides, and plasma AIP were significantly higher in patients, whereas HDL cholesterol levels were also higher compared with controls (all *p* < 0.05). cIMT values were greater in patients (0.45 ± 0.10 mm vs. 0.35 ± 0.00 mm, *p* = 0.001), and hsCRP levels were modestly but significantly elevated (*p* = 0.045).

Measures of insulin sensitivity are presented in Table 3. All eGDR indices (BMI-, WC-, and WHR-based) were markedly lower in patients than in controls (all *p* < 0.001), indicating reduced insulin sensitivity. Both the cardiometabolic index (CMI) and body shape index were significantly higher in the T1DM cohort (*p* < 0.001), supporting the presence of more pronounced metabolic risk.

**Figure 1:**
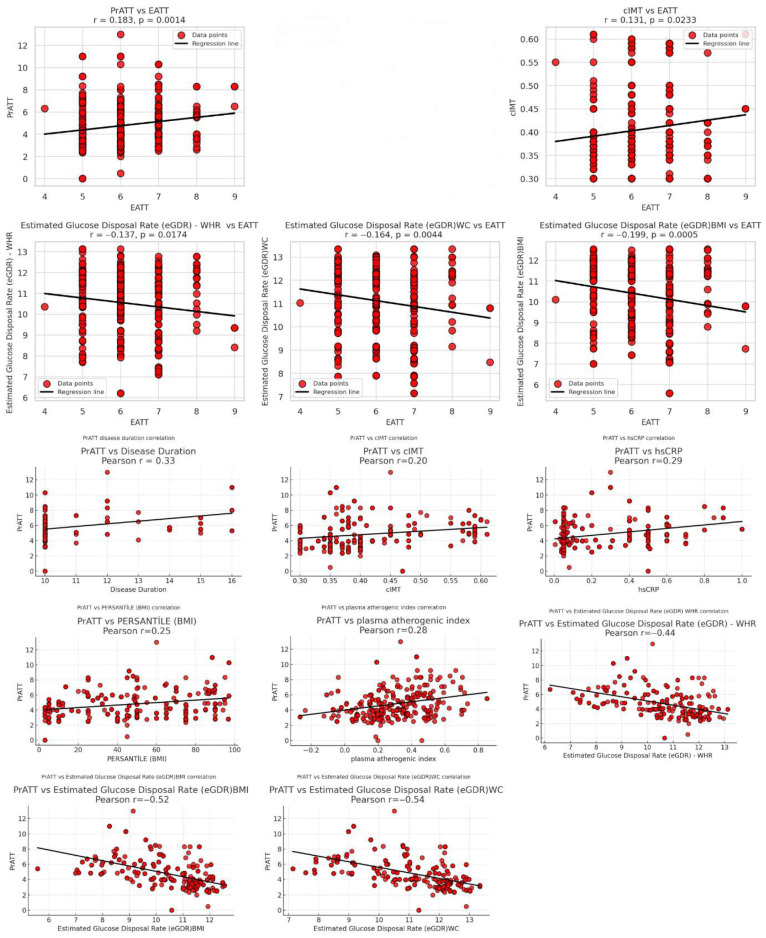
All correlation analyses related to PrATT and EATT.

Correlation analyses linking PrATT with metabolic and anthropometric parameters are shown in Table 4. PrATT demonstrated strong negative correlations with all eGDR indices (r = −0.439 to –0.539, all *p* < 0.001). Perirenal adipose tissue thickness demonstrated moderate, statistically significant positive associations with hepatic steatosis (r = 0.521), as well as with the cardiometabolic index, high-sensitivity C-reactive protein levels, waist and hip circumferences, total cholesterol, the plasma atherogenic index, disease duration, and several other metabolic parameters (all *p* < 0.001). Weak but statistically significant correlations were present with cIMT, age, body shape index, body surface area, WHtR, LDL cholesterol, EATT, and triglycerides. As summarized in Table 5, EATT showed weak but significant correlations with multiple metabolic indicators. EATT was positively correlated with PrATT (*p* = 0.0014), systolic blood pressure, and cIMT (both *p* < 0.05). All eGDR indices were negatively correlated with EATT (*p* < 0.05), reinforcing the association between visceral fat depots and reduced insulin sensitivity. Correlation analyses related to glycemic exposure are presented in the corresponding table. Mean HbA1c showed a strong negative correlation with eGDR-BMI (r = −0.801, *p* < 0.001) and a strong positive association with cIMT (r = 0.577, *p* < 0.001). Table 6 Glycemic load was weakly but significantly correlated with WHR (*p* = 0.037). The results of all correlation analyses are summarized and presented in Figure 1.

**Table 6:** Results of Correlation Analyses Related to mean-HgbA1c and Glucose Burden.

		r	*p*
eGDR (BMI)	mean-HgbA1c	–0.801	<0.001
cIMT	mean-HgbA1c	0.577	<0.001
waist/hip ratio	glucose burden	0.163	0.037

Logistic regression analyses identified several significant independent predictors of case status (Table 7 and Table 8). In multivariable models, PrATT (OR 2.47, *p* < 0.001), EATT (OR 3.41, *p* < 0.001), hsCRP (OR 6202, *p* < 0.001), and cIMT (OR 90.0, *p* < 0.001) remained robust predictors. Among insulin sensitivity indices, eGDR-BMI showed the strongest independent association (OR 0.046, *p* < 0.001), while eGDR-WHR also retained significance (OR 0.412, *p* = 0.011). The body shape index remained statistically significant in the multivariable model (*p* = 0.023), although with an extremely large effect estimate, indicating that these results should be interpreted cautiously and may reflect scaling effects.

A subgroup analysis of patients stratified by BMI percentile (<85th vs. ≥85th) is presented in Table 9. Adolescents with BMI ≥85th percentile had significantly higher weight, BMI, waist circumference, hip circumference, WHtR, WHR, and body shape index (all *p* < 0.0001). Visceral adiposity was more pronounced in this subgroup: both PrATT and hepatic steatosis grade were significantly higher (*p* = 0.001 for both). Disease duration, fasting blood glucose, and glycemic load did not differ significantly between subgroups. However, insulin sensitivity indices were markedly lower in the ≥85th percentile group across all eGDR measures (*p* < 0.0001), and CMI was significantly elevated (*p* = 0.004), indicating clustering of metabolic risk factors in adolescents with higher BMI.

## 4. Discussion

In this cross-sectional study, children and adolescents with T1DM exhibited distinct alterations in visceral adiposity, insulin sensitivity, and cardiometabolic risk compared with healthy controls. Ultrasonographic evaluation revealed that both epicardial and perirenal adipose tissue thicknesses were significantly increased in patients. When the entire cohort was considered, epicardial adipose tissue thickness was consistently greater among participants with type 1 diabetes mellitus compared with healthy controls, [19,20,21,22]. Subgroup analyses by obesity status should be interpreted cautiously due to limited sample sizes and should be confirmed in larger cohorts. These findings suggest that expansion of visceral adipose depots in T1DM may occur partly independently of overall adiposity, reflecting underlying diabetes-related metabolic dysregulation.

All eGDR indices were markedly reduced in the T1DM group, indicating diminished insulin sensitivity that paralleled the increase in visceral adipose accumulation. The inverse correlations between eGDR and both epicardial and perirenal adipose tissue thickness underscore a strong association between insulin resistance and visceral fat deposition in pediatric T1DM. Plasma AIP was significantly elevated in patients, indicating a more atherogenic lipid profile despite higher HDL levels in the T1DM group. This mixed lipid pattern may contribute to early atherosclerotic risk in childhood diabetes. Moreover, patients exhibited slightly elevated yet statistically significant hsCRP concentrations, suggesting the presence of an underlying low-grade inflammatory state that may contribute to early vascular impairment.

Both PrATT and EATT were positively correlated with cIMT, suggesting a link between visceral adiposity and early vascular changes rather than an isolated fat accumulation. Collectively, these findings indicate that even in childhood, T1DM is associated with ultrasonographically detectable alterations in visceral fat distribution, insulin sensitivity, and lipid metabolism, accompanied by early indicators of subclinical atherosclerosis. These observations emphasize the importance of comprehensive cardiometabolic assessment, extending beyond glycemic control, in pediatric patients with T1DM.

The heightened burden of cardiovascular morbidity observed in individuals with type 1 diabetes mellitus has been widely linked to the coexistence of persistent low-grade inflammation and systemic insulin resistance [23,24]. Within this pathophysiological framework, epicardial adipose tissue has emerged as a potentially important contributor to diabetes-related cardiovascular injury, particularly in the presence of increased epicardial fat thickness [25]. Under normal metabolic conditions, epicardial fat functions as a readily available energy source and provides mechanical cushioning for the myocardium [26]. In contrast, chronic hyperglycemia and relative insulin deficiency in diabetes induce both structural remodeling and functional disturbances within this adipose depot, resulting in altered secretory behavior [27,28]. This maladaptive shift is characterized by an excess release of proinflammatory and proatherogenic adipokines alongside a relative reduction in anti-inflammatory mediators, thereby facilitating local vascular inflammation and endothelial dysfunction [27,28,29,30]. The higher EATT observed in our T1DM cohort is consistent with prior pediatric studies [19,20,21,22] and supports the concept that increased epicardial fat may represent an early, subclinical factor that heightens susceptibility to atherosclerotic cardiovascular disease in children with T1DM.

A further noteworthy finding of the present study concerns PrATT. PrATT levels were significantly elevated in both obese and non-obese children with T1DM compared with healthy controls, with greater values among those in the ≥85th BMI percentile. Moreover, PrATT showed moderate correlations with hepatic steatosis, hsCRP, and cIMT, suggesting that perirenal fat may function as a sensitive indicator of early metabolic stress even in the absence of overt obesity. Previous studies have identified PrATT as an important cardiometabolic marker in adults [31,32,33]. Our data extend this evidence to the pediatric T1DM population and support the notion that early perirenal adipose tissue accumulation may form part of the pathophysiological continuum linking T1DM with increased cardiometabolic risk.

Emerging evidence indicates that visceral adipose tissue exerts vascular effects that precede overt atherosclerosis. Perirenal and epicardial depots release adipokines, cytokines, and free fatty acids that promote endothelial activation, oxidative stress, and vascular remodeling [34,35,36]. These mechanisms contribute to intimal thickening and decreased arterial compliance, hallmarks of early atherogenesis. Consistent with these mechanisms, both PrATT and EATT in our cohort were positively correlated with cIMT and hsCRP, highlighting their potential contribution to early vascular remodeling. Notably, increased visceral adiposity and elevated cIMT were present despite comparable BMI values between groups, underscoring the limitation of BMI as a standalone cardiometabolic risk indicator. This finding aligns with previous reports of increased visceral adiposity and arterial stiffness in normal-weight children with T1DM [37,38,39] and underscores the importance of imaging-based assessments for early detection of vascular risk.

Although T1DM is classically defined by β-cell destruction and absolute insulin deficiency, increasing evidence indicates that insulin resistance coexists and modulates disease progression and vascular risk in many patients [23,24]. The concept of “double diabetes,” in which patients with T1DM develop features of insulin resistance typical of type 2 diabetes, is particularly relevant in adolescents [40,41]. In this setting, excess visceral fat acts as an active metabolic compartment that secretes lipotoxic and inflammatory factors capable of driving both metabolic impairment and endothelial dysfunction [42,43]. The present findings of increased PrATT and EATT with concurrent reductions in eGDR support an association—rather than a proven causal relationship—between visceral fat accumulation and reduced insulin sensitivity in pediatric T1DM.

The positive correlations between hepatic steatosis, PrATT, and reduced eGDR further underscore shared pathophysiological mechanisms linking ectopic fat accumulation and vascular dysfunction. Hepatic fat deposition represents an additional manifestation of insulin resistance and has been associated with impaired endothelial function and increased carotid stiffness in both T1DM and type 2 diabetes [44]. The coexistence of elevated hepatic steatosis, visceral adiposity, and inflammation in our cohort supports the concept of a multisystem “pre-atherosclerotic” state in pediatric diabetes.

Parallel to these alterations in visceral adiposity and early vascular changes, AIP was evaluated as an additional indicator of dyslipidemia and cardiovascular risk. The atherogenic index of plasma was markedly increased across the pediatric type 1 diabetes cohort and tended to be higher among participants with excess body weight; however, the sample size was insufficient to support robust subgroup-specific inferences. The modest correlation between AIP and glycemic exposure supports a potential interaction between glucose dysregulation and lipid metabolism. In line with previous meta-analyses demonstrating a more atherogenic lipid profile in pediatric T1DM populations, our findings suggest that AIP may serve as a practical and accessible biomarker for early cardiometabolic risk assessment [45].

In logistic regression analyses, PrATT, EATT, hsCRP, and cIMT emerged as independent predictors of T1DM status, whereas lower eGDR values were strongly associated with case status. These results highlight the independent contribution of visceral adiposity and inflammation to early vascular alterations and underscore their potential utility as clinical screening markers in pediatric diabetes. However, the very large effect estimates observed for several variables indicate that further work is needed to optimize model specification and scaling, and that these models should be validated in independent cohorts before clinical application.

Finally, CMI was significantly increased among children with T1DM. As a composite indicator derived from WHtR and the triglyceride-to-HDL cholesterol ratio, CMI reflects the combined burden of visceral fat accumulation and dyslipidemia [46,47]. Recent large-scale studies have shown that elevated CMI is strongly associated with insulin resistance, metabolic-associated fatty liver disease, and increased cardiovascular risk [47,48,49,50]. The rise in CMI observed in our cohort reinforces the presence of early metabolic dysregulation in pediatric T1DM and supports its potential utility as a noninvasive marker of cardiometabolic risk in this population.

## 5. Limitations and Conclusions

This study has several limitations. First, its cross-sectional design precludes causal inferences between visceral adiposity and vascular or metabolic outcomes. Second, eGDR is a surrogate index of insulin sensitivity rather than a direct measure such as the euglycemic–hyperinsulinemic clamp. Third, subgroup analyses—particularly among participants with BMI ≥85th percentile—included relatively small numbers, limiting statistical power. Fourth, quantification of visceral fat depots was based on linear thickness measurements; volumetric imaging (MRI or CT) would provide greater precision but is less feasible in routine pediatric practice. Fifth, logistic regression analyses were constrained by sample size and by the scaling of continuous predictors, which may have contributed to very large effect estimates for some variables. Finally, residual confounding from lifestyle factors (diet, physical activity, and insulin regimen) cannot be excluded.

In conclusion, ultrasonographically measured perirenal and epicardial adipose tissue thicknesses were increased in children and adolescents with T1DM and were associated with insulin resistance, systemic inflammation, and subclinical atherosclerosis as indicated by elevated cIMT. These associations suggest that visceral adiposity may contribute to early vascular dysfunction in pediatric diabetes, even in the absence of obesity. Incorporating noninvasive ultrasound markers of visceral fat into clinical assessment may facilitate early identification of children at high cardiovascular risk and help guide timely preventive interventions. Prospective, longitudinal studies using standardized imaging and direct measures of insulin sensitivity are needed to clarify causal pathways and to refine risk stratification in this vulnerable population.

## Figures and Tables

**Table 1:** Demographic and Anthropometric Findings.

	Patient (n = 150) Mean ± SD	Control (n = 152) Mean ± SD	*p* Value
Gender Male ** (n, %) Female	86 (%54.3) 64 (%42.6)	72 (%47.3) 80 (%52.6)	0.106
Age * (yıl)	16.33 ± 2.73	15.92 ± 2.79	0.201
mean-HgbA1c	8.62 ± 1.55	-	
Disease Duration	11.07 ± 1.91	-	
Glucose Exposure	287.57 ± 202.67	-	
Weight ** (kg)	56.67 ± 9.10	54.46 ± 6.91	0.081
Height ** (cm)	166.27 ± 8.34	165.55 ± 8.89	0.273
Body surface area * (m^2^)	1.62 ± 0.18	1.56 ± 0.20	0.143
Body Mass Index * (kg/m^2^)	20.41 ± 3.29	19.89 ± 2.69	0.132
Body Mass Index * (percentile)	51.73 ± 29.68	45.00 ± 29.89	0.052
Waist circumference * (cm)	70.82 ± 9.32	68.39 ± 7.89	***0.010***
Hip circumference * (cm)	87.54 ± 7.62	85.24 ± 6.60	***0.003***
Waist/Hip Ratio *	0.81 ± 0.07	0.80 ± 0.08	0.136
Waist/Height Ratio *	0.43 ± 0.07	0.41 ± 0.06	0.181
Diastolic Blood Pressure * (mmHg)	69.2 ± 5.45	68.0 ± 5.5	0.152
Systolic Blood Pressure * (mmHg)	115.87 ± 9.49	114.23 ± 9.50	0.062

* Mann–Whitney U ** Pearson χ^2^.

**Table 2:** Visceral Adiposity and Cardiometabolic Findings.

	Patient (n = 150) Mean ± SD	Control (n = 152) Mean ± SD	*p* Value
PrATT *	5.89 ± 1.98	3.72 ± 1.21	***<0.001***
EATT * (mm)	6.15 ± 0.91	5.97 ± 0.94	***0.022***
Hepatosteatoz * (grade)	0.39 ± 0.59	0.06 ± 0.26	***<0.001***
Total Cholesterol * (mg/dL)	167.08 ± 26.94	147.95 ± 21.39	***<0.001***
LDL * (mg/dL)	93.34 ± 24.00	83.56 ± 24.22	***0.001***
HDL * (mg/dL)	58.74 ± 13.92	47.19 ± 9.87	***<0.001***
Triglyceride * (mg/dL)	116.39 ± 128.90	90.13 ± 45.98	***0.040***
Plasma Atherogenic Index ***	0.34 ± 0.20	0.24 ± 0.18	***<0.001***
cIMT * (mm)	0.45 ± 0.1	0.35 ± 0.00	***0.001***
hsCRP * (mg/L)	0.35 ± 0.55	0.30 ± 0.15	***0.045***

* Mann–Whitney U, *** *t*-test, PrATT: Perirenal Adipose Tissue Thickness, CMI: Cardiometabolic Index, LDL: low density lipoprotein, HDL: high density lipoprotein, cIMT: carotid intima media thickness, hsCRP: high sensitive C-reactive protein.

**Table 3:** Findings on Insulin Sensitivity.

	Patient (n = 150) Mean ± SD	Control (n = 152) Mean ± SD	*p* Value
eGDR (BMI)	9.28 ± 1.21	11.47 ± 0.59	***<0.001***
eGDR (WC)	10.04 ± 1.23	12.14 ± 0.71	***<0.001***
eGDR (WHR)	9.52 ± 1.24	11.53 ± 0.95	***<0.001***
Cardiometabolic Index	0.46 ± 0.22	0.35 ± 0.16	***<0.001***
Body Shape Index	0.0737 ± 0.0050	0.0716 ± 0.0040	***<0.001***

eGDR: Estimated Glucose Disposal Rate, BMI: body mass index, WC: waist circumference, WHR: waist-to-heigt ratio, CMI: cardiometabolic index, BSI: body shape index.

**Table 4:** Results of Correlation Analyses Related to PrATT.

	r (Pearson)	*p* Value
eGDR (WC)	−0.539	*p* < 0.001
eGDR (BMI)	−0.519	*p* < 0.001
eGDR (WHR)	−0.439	*p* < 0.001
Hepatosteatoz	0.521	*p* < 0.001
CMI	0.328	*p* < 0001
hsCRP	0.292	*p* < 0.0001
Waist Circumference	0.363	*p* < 0.001
Total Cholesterol	0.350	*p* < 0.001
Hip Circumference	0.372	*p* < 0.001
Plasma atherogenic index	0.275	*p* < 0.001
Disease Duration	0.335	*p* < 0.001
cIMT	0.201	*p* = 0.0015
Age	0.261	*p* < 0.001
Body shape index	0.275	*p* < 0.001
Body Surface Area	0.250	*p* < 0.001
Weist/Height ratio	0.304	*p* < 0.001
LDL	0.189	*p* = 0.010
EATT	0.183	*p* = 0.014
Triglyceride	0.245	*p* < 0.001

**Table 5:** Results of Correlation Analyses Related to EATT.

	R (Pearson)	*p* Value
PrATT	0.183	0.0014
cIMT	0.131	0.0233
eGDR-WHR	−0.137	0.0174
eGDR-WC	−0.164	0.0044
eGDR-BMI	−0.199	0.0005

**Table 7:** Logistic Regression Analysis, part 1.

	Univ. β	OR	95% CI	*p*	Multiv. β	OR	95% CI	*p*
PrATT, (mm)	0.98	2.67	2.11–3.37	***<0.001***	0.90	2.47	1.76–3.46	***<0.001***
EATT, (mm)	0.57	1.77	1.36–2.29	***<0.001***	1.23	3.41	1.81–6.43	***<0.001***
hsCRP (mg/L)	7.22	1369	248–7542	***<0.001***	8.73	6202	288–133 710	***<0.001***
cIMT (per 0.1 mm)	3.441	31.20	12.53–77.68	***<0.001***	4.500	90.00	24.47–331.01	***<0.001***
Plasma Atherogenic Index	2.84	17.1	4.79–60.95	***<0.001***	-	-	-	-
Total Kolesterol (mg/dL)	0.03	1.03	1.02–1.05	***<0.001***	-	-	-	-
LDL (mg/dL)	0.02	1.02	1.01–1.04	***0.008***	–	–	–	–
HDL (mg/dL)	–0.04	0.96	0.94–0.98	***<0.001***	–	–	–	–
Trigliserid (mg/dL)	0.004	1.004	1.001–1.007	***0.019***	–	–	–	–
Bel çevresi (cm)	0.07	1.07	1.05–1.10	***<0.001***	-	-	-	-
Kalça çevresi (cm)	0.05	1.05	1.03–1.07	***<0.001***	–	–	–	–
Waist/Hip Ratio	5.12	167	16.6–1670	***<0.001***	–	–	–	–
BMI (kg/m^2^)	0.11	1.12	1.06–1.19	***<0.001***	–	–	–	–
Body Shape Index	95.6	2.0 × 10^41^	1.4 × 10^12^–3.0 × 10^69^	***<0.001***	–	–	–	–

PrATT: Perirenal Adipose Tissue Thickness, cIMT: carotid intima media thickness.

**Table 8:** Logistic regression analysis, part 2.

	Univ β	OR	OR 95% CI	*p*	Multiv β	OR	OR 95% CI	*p*
eGDR (BMI)	–2.93	0.053	[0.028–0.103]	***<0.001***	–3.08	0.046	[0.015–0.137]	***<0.001***
eGDR (WC)	–2.26	0.104	[0.063–0.171]	***<0.001***	-	-	-	**-**
eGDR (WHR)	–1.67	0.188	[0.130–0.273]	***<0.001***	–0.89	0.412	[0.207–0.819]	***0.011***
Cardiometabolic Index	3.22	24.94	[6.247–99.609]	***<0.001***	-	-	-	**-**
Body Shape Index	106.89	~2.63 × 10^46^	[6.47 × 10^23^–1.07 × 10^69^]	***<0.001***	–136.93	≈0	[0.000–0.000]	***0.023***

eGDR: Estimated Glucose Disposal Rate.

**Table 9:** A Subgroup Analysis in Patients According to BMI (percantil).

	<85 Mean ± SD (Median) (n = 111)	≥85 Mean ± SD (Median) (n = 39)	*p*-Value
Gender (n) Female Male	41 70	23 16	*0.027*
Age, (year)	16.14 ± 2.66 (16.00)	16.87 ± 2.88 (18.00)	0.1210
Weight, (kg)	54.95 ± 8.90 (54.00)	61.69 ± 7.82 (63.00)	***<0.0001***
Height, (cm)	168.65 ± 7.32 (168.00)	159.49 ± 7.32 (162.00)	***<0.0001***
Body Mass Index	19.01 ± 2.45 (18.70)	24.37 ± 1.81 (24.00)	***<0.0001***
Body Surface Area, (m^2^)	1.61 ± 0.18 (1.60)	1.64 ± 0.17 (1.62)	0.3220
Hip Circumference, (cm)	85.71 ± 6.97 (88.00)	92.74 ± 7.01 (96.00)	***<0.0001***
Waist Circumference, (cm)	66.79 ± 6.53 (67.00)	82.31 ± 5.81 (83.00)	***<0.0001***
Waist/Height ratio	0.40 ± 0.04 (0.39)	0.52 ± 0.04 (0.51)	***<0.0001***
Waist/Hip ratio	0.78 ± 0.05 (0.78)	0.89 ± 0.05 (0.89)	***<0.0001***
Body Shape Index	0.07 ± 0.01 (0.07)	0.08 ± 0.00 (0.08)	***<0.0001***
Diastolic BP, (mmHg)	68.1 ± 5.45	68.0 ± 5.0	0.451
Systolic BP, (mmHg)	115.75 ± 8.40	115.25 ± 10.50	0.150
EATT, (mm)	6.42 ± 0.93 (6.00)	6.15 ± 0.81 (6.00)	0.1760
PrATT, (mm)	5.56 ± 1.81 (5.40)	6.83 ± 2.17 (6.30)	***0.001***
Hepatosteatoz, (grade)	0.30 ± 0.53 (0.00)	0.67 ± 0.66 (1.00)	***0.001***
Disease Duration, (year)	10.98 ± 1.81 (10.00)	11.33 ± 2.16 (10.00)	0.3260
FBG, (mg/dL)	196.44 ± 84.59 (202.00)	202.44 ± 96.47 (244.00)	0.3390
Glucose Exposure	278.53 ± 187.69 (244.00)	313.31 ± 241.16 (230.00)	0.5940
Total Cholesterol, (mg/dL)	165.50 ± 25.31 (166.50)	171.57 ± 31.04 (161.00)	0.4560
LDL, (mg/dL)	92.16 ± 22.95 (91.90)	96.70 ± 26.81 (94.50)	0.6150
HDL, (mg/dL)	60.09 ± 15.40 (57.70)	54.92 ± 7.28 (55.90)	0.0980
Triglyceride, (mg/dL)	109.05 ± 85.45 (87.00)	137.31 ± 208.35 (93.60)	0.1330
Plasma Atherogenic Index	0.35 ± 0.22 (0.35)	0.34 ± 0.16 (0.37)	0.5070
hsCRP, (mg/L)	0.43 ± 0.27 (0.50)	0.28 ± 0.19 (0.30)	***0.001***
cIMT, (mm)	0.45 ± 0.08 (0.45)	0.46 ± 0.10 (0.45)	0.9950
eGDR-WHR	9.98 ± 1.03 (10.21)	8.23 ± 0.82 (8.36)	***<0.0001***
eGDR-WC	10.50 ± 1.03 (10.84)	8.71 ± 0.69 (8.94)	***<0.0001***
eGDR-BMI	9.69 ± 1.03 (9.86)	8.08 ± 0.83 (8.26)	***<0.0001***
Cardiometabolic Index	0.43 ± 0.23 (0.41)	0.52 ± 0.19 (0.49)	***0.004***

Chi-square Test, EATT: Epicardial Adipose Tissue Thickness, eGDR: Estimated Glucose Disposal Rate, Waist/Height Ratio, WC: waist circumference, BMI, body mass index, hsCRP: high sensitive C-reactive protein, FBG: Fasting Blood Glucose.

## Data Availability

The data presented in this study are available on request from the corresponding author. The data are not publicly available due to privacy and ethical reasons.

## Abbreviations

AIP	Atherogenic Index of Plasma
ASE	American Society of Echocardiography
BMI	Body Mass Index
BSA	Body Surface Area
cIMT	Carotid Intima–Media Thickness
CMI	Cardiometabolic Index
CT	Computed Tomography
EAT	Epicardial Adipose Tissue
EATT	Epicardial Adipose Tissue Thickness
ECG	Electrocardiogram
eGDR	Estimated Glucose Disposal Rate
HbA1c	Hemoglobin A1c
HC	Hip Circumference
HDL	High-Density Lipoprotein
hsCRP	High-Sensitivity C-Reactive Protein
HTN	Hypertension
IQR	Interquartile Range
ISPAD	International Society for Pediatric and Adolescent Diabetes
LDL	Low-Density Lipoprotein
MRI	Magnetic Resonance Imaging
PRAT	Perirenal Adipose Tissue
PrATT	Perirenal Adipose Tissue Thickness
SARS-CoV-2	Severe Acute Respiratory Syndrome Coronavirus 2
SD	Standard Deviation
SE	Standard Error
T1DM	Type 1 Diabetes Mellitus
TG	Triglyceride
TTE	Transthoracic Echocardiography
WC	Waist Circumference
WHR	Waist-to-Hip Ratio
WHtR	Waist-to-Height Ratio
